# Update on HIV reservoirs

**DOI:** 10.1186/1471-2334-14-S2-S18

**Published:** 2014-05-23

**Authors:** Liang Shan

**Affiliations:** 1 John Hopkins University, Baltimore, Maryland, 21218-2683, USA

## 

The stable latent reservoir for HIV-1 in resting memory CD4^+^ T cells is a major barrier to curing HIV-1/AIDS. Studies on HIV-1 latency focused on regulation and reactivation of viral gene expression in the cells in which viral latent infection is already established. However, it is not well understood how virus becomes latent. In this study, we found that in a subset of memory precursor CD4^+^ T cells, HIV-1 completes steps in the life cycle through integration but has limited gene transcriptional activity. HIV-1 preferentially establishes latent infection in this subset of CD4^+^ T cells. Other CD4^+^ T cells are not latency permissive because they either block viral entry/reverse transcription, or support high level viral gene transcription. We also investigated how CD8^+^ T cells could affect HIV-1 latent infection. We found that viral-specific CTLs could effectively block or clear HIV-1 latent infection. Since immunodominant CTL responses in acute infection have been identified as the major selection force driving the development of CTL escape mutations, we hypothesized that patient latent viruses should carry mutated epitopes which could not be recognized by immunodominant CTLs. In fact, we confirmed that the vast majority (>98%) of latent viruses carried CTL escape mutations that render the infected cells insensitive to immunodominant CTLs. To solve this critical problem, we identified CTLs that could recognize antigens from latent HIV-1 that were unmutated in chronically infected patient. We further demonstrated that these CTLs were able to eliminate target cells infected with autologous virus derived from the latent reservoir. The predominance of CTL-resistant viruses in the latent reservoir poses a major challenge to viral eradication. Our results demonstrate that HIV-1 establishes latent infection in memory precursor CD4^+^ T cells. Chronically infected patients retain a broad spectrum viral specific CTL response and that appropriate boosting of this response may be required for the elimination of the latent reservoir.

